# Anaphylaxis triggered by prick test with latex extract: a case report

**DOI:** 10.1590/1516-3180.2017.0295031217

**Published:** 2018-06-11

**Authors:** Leonardo Oliveira Mendonça, Cynthia Mafra Fonseca de Lima, Priscilla Rios Cordeiro Macedo, Victor do Espirito Santo Cunha, Ruppert Ludwig Hahnstadt, Jorge Kalil, Clóvis Eduardo Santos Galvão

**Affiliations:** I MD. Postgraduate Fellow, Clinical Immunology and Allergy Division, Faculdade de Medicina da Universidade de São Paulo (FMUSP), São Paulo (SP), Brazil; II MD, MSc. Physician and Doctoral Student, Clinical Immunology and Allergy Division, Faculdade de Medicina da Universidade de São Paulo (FMUSP), São Paulo (SP), Brazil; III MD. Physician and Master’s Student, Clinical Immunology and Allergy Division, Faculdade de Medicina da Universidade de São Paulo (FMUSP), São Paulo (SP), Brazil; IV MSc, PhD. Veterinarian and Research & Development Manager, Immunotech Laboratories, FDA-Allergenics, Rio de Janeiro (RJ), Brazil; V Pharm, MSc. Pharmacist and Head Director, Immunotech Laboratories, FDA-Allergenics, Rio de Janeiro (RJ), Brazil; VI MD, PhD. Full Professor, Clinical Immunology and Allergy Division, Hospital das Clínicas, Faculdade de Medicina da Universidade de São Paulo (FMUSP), São Paulo (SP), Brazil; VII MD, PhD. Attending Physician, Clinical Immunology and Allergy Division, Hospital das Clínicas, Faculdade de Medicina da Universidade de São Paulo (FMUSP), São Paulo (SP), Brazil

**Keywords:** Latex hypersensitivity, Diagnostic test, routine, Anaphylaxis

## Abstract

**CONTEXT:**

Adverse reactions associated with prick tests are rare but may be present as serious systemic reactions.

**CASE REPORT:**

A 38-year-old female nursing technician complained of three episodes of anaphylaxis in one year, all in the workplace. To investigate latex allergy, the patient underwent the prick test with latex, and immediately developed a rash, itchy skin, hoarseness, dyspnea and dry cough. Her condition improved promptly after appropriate measures were established for controlling her anaphylaxis.

**CONCLUSION:**

The skin test must be performed under medical supervision, since complications that can lead to life-threatening reactions, if support measures are not readily implemented, have been attributed to this test.

## INTRODUCTION

Anaphylaxis is a well-recognized clinical syndrome caused by the pharmacological activity of mediators that are released after activation of mast cells and basophils through contact with an antigen. It is a serious systemic reaction with an acute onset and it is potentially fatal.^1^ Specialists need to be able to recognize and treat this syndrome and to be aware of both the unpredictability of its onset and the potential for severe outcomes through its evolution.

The specific diagnostic and/or therapeutic activities of immunological allergy outpatient clinics, such as administration of allergen-specific immunotherapy, challenge tests and skin tests for immediate hypersensitivity to allergens, may lead to appearance of systemic allergic reactions that can be serious and even fatal. The incidence of systemic reactions to skin tests for immediate hypersensitivity is small, and the test is considered safe. Nonetheless, the possibility of systemic reactions should not be underestimated, especially in relation to extracts of Hymenoptera venoms and medications.^2^

In clinical practice, investigations on sensitivity are conducted initially through *in vitro* sensitization tests or skin prick tests. Both of these methods are considered safe. In *in vitro* tests, intercurrences are very rare and, when they do occur, they are due to complications during blood collection. Reactions relating to prick tests are also rare and may be due to the procedure itself (puncture wounds) or to serious reactions that might be triggered because of exposure to the allergen.^3^

Occupational anaphylaxis has become more prominent through recognition that this serious condition may occur in occupational contexts. Among the occupational agents, latex has gained prominence over recent years, especially among healthcare professionals, because of the severity of the reactions^4^.

This paper aimed to describe a case of anaphylaxis triggered by a prick test with latex extract, in a patient with occupational anaphylaxis. Until now, no case of anaphylaxis during a skin test with latex extract had been reported in Brazil.

## CASE REPORT

A 38-year-old female nursing technician presented a prior history of three episodes of anaphylaxis within one year, all in the workplace. She had a personal history of mild intermittent allergic rhinitis from childhood, for which antihistamines and nasal corticosteroid had been used only during exacerbated episodes. She had a family history of atopy and presented a positive prick test for aeroallergens (*Dermatophagoides pteronyssinus* and *Blomia tropicalis*). She tested negative for serum-specific immunoglobulin E (IgE) for latex.

Because of her recurrent pattern of anaphylaxis and risk factor for latex allergy, a latex prick test using a standard commercial extract (500 mcg/ml; ALK Abelló, Spain) was performed. Five minutes after the puncturing, the patient developed a generalized rash, itchy skin, hoarseness, dyspnea, dry cough and a sensation of a foreign body in the oropharynx. Her vital signs were: blood pressure of 134 x 84 mmHg, heart rate of 130 bpm and peripheral oxygen saturation of 94%. The patient was placed in dorsal decubitus, with elevation of the lower limbs and 0.5 mg of adrenaline was applied intramuscularly in the upper third of the vastus lateralis muscle of the thigh, in addition to 200 mg of hydrocortisone and 50 mg of diphenhydramine intravenously, and inhalation of short-acting ß2-agonist. The patient presented progressive improvement of the condition without presentation of the late-phase reaction.

The patient was evaluated in the context of another major study that was ongoing, and signed a free and informed consent statement for that study, which had been approved by the institution’s ethics committee, under the number 0538/10 on September 22, 2010.

## DISCUSSION

Immediate skin prick tests are a useful tool for assessing IgEmediated sensitization, to make an etiological diagnosis for allergic manifestations of Gell and Coombs type I reactions. They have been performed within the clinical practice of allergist physicians for many years and are considered extremely safe.

In 1987, Lockey et al.^3^ published a report on cases of fatalities during immunotherapy and skin tests for mites. These authors reviewed the mortality cases that occurred between 1973 and 1983 through medical contacts in the United States. Up until that time, only six deaths through skin tests had been reported. Of these, four cases were male and two were children under 18 years. Almost all the patients (five) had asthma and were not currently being treated.

In 1993, Reid et al.^5^ reviewed the cases of mortality due to immunotherapy and skin tests for mites in the United States that occurred between 1985 and 1989. A total of 13 deaths were reported during this period, none of them related to the skin prick test. The vast majority of the patients were asthmatic patients undergoing treatment, and mortality was attributed to the severity of the underlying allergic disease as well as associations with other medications that are known to aggravate anaphylactic reactions, such as beta-blockers. At that time in the United States, 33 million doses of immunotherapeutic drugs were being used per year, and there was a proportion of 1 fatal reaction for every 2 million doses applied.

In the most recent survey, published in 2004, Bernstein et al.^6^ reviewed all fatalities consequent to prick tests and immunotherapy in the United States and Canada from 1990 to 2001. In their study, aeroallergens and foods were evaluated and it was found that a total of 19 deaths occurred due to immunotherapy and a single death occurred during a skin prick test. This last death occurred in a hospital environment during application of multiple food allergens.

The initial step in diagnosing latex allergy is to obtain a thorough clinical history. However, to confirm the etiology, it is necessary to determine the presence of specific IgE *in vitro* (serological tests) or *in vivo* (skin prick tests). Serological tests have a high number of false negative results and should not be used alone for screening of latex allergy.^4^ This may happen because not all latex allergens are represented in the assay, and it may explain why the serological test was negative in the case presented here. Skin prick tests have higher specificity and sensitivity than do *in vitro* tests,^4^ and should be used whenever possible, although they have been associated with anaphylactic events, as presented here. In this case, because we were facing a probable occupational case with its labor-law implications, we decided to continue with the etiological investigation and carry out the prick test.

In clinical practice, the first safety measure that should be established is removal of the causal agent, which in this case meant replacement of latex with latex-free products. In addition, specific allergen immunotherapy should be implemented, but this is not available in Brazil.

We reviewed the literature in MEDLINE, PubMed and LILACS using the English-language keywords “latex hypersensitivity”, “diagnostic test” and “anaphylaxis”; the Portuguese keywords “hiper-sensibilidade ao latex”, “teste diagnóstico” and “anafilaxia”; and the Spanish keywords “hipersensibilidad al látex”, “prueba de diagnóstico” and “anafilaxia”. We did not find any case report describing an anaphylaxis reaction during a skin prick test with latex (**Table 1**).

**Table 1.** Search of the medical literature for case reports on adverse reactions associated with diagnostic test for latex allergy. The search was conducted on July 5, 2017

Database	Search strategies	Papers found	Related papers
MEDLINE (via PubMed)	(Latex hypersensitivity AND Diagnostic test) or (Latex Hypersensitivity AND Anaphylaxis) or (Latex hypersensitivity AND Diagnostic test AND Anaphylaxis)?, in ALL FIELDS	23	3
LILACS (via BIREME)	(Latex hypersensitivity AND Diagnostic test) or (Latex Hypersensitivity AND Anaphylaxis) or (Latex hypersensitivity AND Diagnostic test AND Anaphylaxis)?, in ALL FIELDS	17	2

## CONCLUSION

The skin prick test for allergen sensitization research is a safe test, but it must be performed in an appropriate environment, under medical supervision and with prior analysis on patients, given that complications have been attributed to this method. These complications may be serious and may lead to death if support measures are not readily available.
